# Eco-evolutionary robustness of wild bacterial communities to experimental perturbation

**DOI:** 10.1093/ismejo/wraf144

**Published:** 2025-07-22

**Authors:** Duhita G Sant, Thomas P Smith, Edgar L Y Wong, Juli Cohen, Kayla C King, Thomas Bell, Timothy G Barraclough

**Affiliations:** Department of Biology, University of Oxford, 11a Mansfield Road, Oxford OX1 3SZ, United Kingdom; Duhita G. Sant, Center for Advanced Biotechnology and Medicine, 679 Hoes Lane West, Piscataway, NJ 08854-8021, United States. Edgar L.Y. Wong, Senckenberg Biodiversity and Climate Research Centre, Frankfurt am Main, Germany; Department of Life Sciences, Imperial College London, Silwood Park Campus, Buckhurst Road, Ascot, Berkshire SL5 7PY, United Kingdom; Department of Biology, University of Oxford, 11a Mansfield Road, Oxford OX1 3SZ, United Kingdom; Duhita G. Sant, Center for Advanced Biotechnology and Medicine, 679 Hoes Lane West, Piscataway, NJ 08854-8021, United States. Edgar L.Y. Wong, Senckenberg Biodiversity and Climate Research Centre, Frankfurt am Main, Germany; Department of Biology, University of Oxford, 11a Mansfield Road, Oxford OX1 3SZ, United Kingdom; Department of Biology, University of Oxford, 11a Mansfield Road, Oxford OX1 3SZ, United Kingdom; Department of Zoology, University of British Columbia, #3051 - 6270 University Blvd, Vancouver, BC V6T 1Z4, Canada; Department of Microbiology & Immunology, University of British Columbia, 2350 Health Sciences Mall #1365, Vancouver, BC V6T 1Z3, Canada; Department of Life Sciences, Imperial College London, Silwood Park Campus, Buckhurst Road, Ascot, Berkshire SL5 7PY, United Kingdom; Department of Biology, University of Oxford, 11a Mansfield Road, Oxford OX1 3SZ, United Kingdom

**Keywords:** tree hole communities, experimental evolution, liming, eco-evolutionary dynamics

## Abstract

Most knowledge about bacterial evolution and ecological interactions comes from laboratory studies. One difference between the wild and most laboratory experiments is the diversity of bacterial taxa present. Understanding how wild bacteria respond to perturbation therefore requires consideration of how ecological sorting, colonization, and genetic changes of constituent species interact. Ecological sorting of species might reduce evolutionary rates and make communities robust to disturbance, or it could amplify selection pressures and lead to unstable co-evolutionary cascades. Even estimates of basic rates of ecological sorting, dispersal, and genetic change are rare. Here, we addressed these knowledge gaps by liming wild decomposer communities living in beech tree holes and tracking ecological and evolutionary responses for 12 weeks. Overall, tree hole communities were extremely robust to liming involving short-term pulses up to 4 pH units and long-term increases up to 2 pH units. Species diversity and composition displayed significant but small changes in treatment tree holes compared to control ones. New bacterial taxa colonized at a low rate that did not vary with liming. Genetic changes in the frequency of single nucleotide polymorphisms in metagenome assembled genomes occurred at rates that were both comparable to and correlated with ecological changes in the same metagenome assembled genomes, but the rate of genetic changes did not vary between limed and control tree holes. Analysis of rates of genetic change estimated low effective population size (~10^4^) and generation times of roughly 1 day. Our study provides estimates of rates of ecological and evolutionary processes in wild bacterial communities, which displayed remarkable robustness to our experimental perturbation.

## Introduction

All species live in diverse assemblages with many hundreds of other species. A key challenge is to understand complex ecosystems in enough detail to predict how they will respond to changing environments [1]. This is difficult because whole-system responses depend on traits of all constituent species and the interactions among them [2]. Furthermore, species traits can evolve over time, and the way that species evolve (both process and outcome) can both affect and be affected by ecological changes in abundance and distribution of the surrounding community [3, 4]. If ecological and evolutionary dynamics operate over similar timescales, eco-evolutionary feedbacks can occur [5]. For example, a recent experiment showed that ecological and evolutionary processes together drive changes in siderophore production in bacterial communities facing copper stress [6]. Accounting for both ecological and evolutionary responses is therefore vital for understanding how diverse communities respond to environmental change [7, 8].

Understanding ecological and evolutionary responses is particularly important in bacteria, which perform many processes that human populations depend upon, such as global nutrient cycling, decomposition, and bioremediation [9, 10]. Bacteria live in highly diverse communities of hundreds or thousands of species (where “species” refers to genetically and ecologically discrete strains that co-occur in sympatry) and can disperse widely via resistant stages such as spores. There may be a vast pool of functionally diverse species able to respond to any environmental change, resulting in rapid shifts in community composition [11, 12]. Cataloguing bacterial diversity has been transformed by DNA sequencing, but few studies track temporal dynamics in communities [13–15] and even fewer look at responses to experimental perturbations [16, 17]. Simple questions like how fast do species abundances fluctuate over time? and what is the role of the influx of new species by dispersal versus local dynamics within habitat patches? remain under-explored.

Bacteria have the potential for rapid evolution thanks to their short generation time, vast population sizes, and an array of mechanisms for generating genetic variation. It might be expected therefore that environmental change would trigger evolution over similar timescales to ecological changes. Most knowledge of bacterial evolution comes from the laboratory, however, and mostly with single species exposed to strong selection pressures such as antibiotics [18, 19] and parasite phage [20]. Species interactions have been shown to alter the trajectory and magnitude of evolution in simple community microcosms in the laboratory [20–25], but we still know little about how bacteria evolve in the wild. It is unclear whether bacteria evolve as rapidly as implied by experimental evolution, or whether evolution is constrained by factors such as slower generation times [4], ecological changes in species abundances [26], trade-offs between multiple selection pressures such as competitors [27] or phages [28], and frequent dispersal between environments that might preclude specialization on any particular habitat [29].

**Figure 1:**
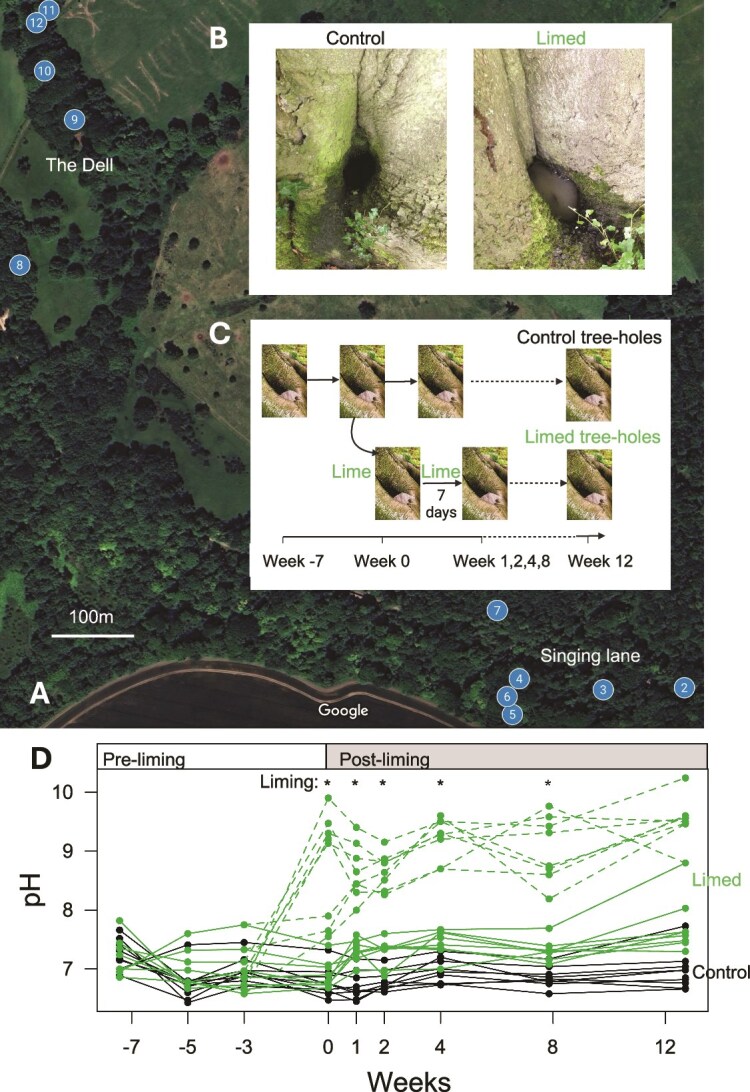
(**A)** Location of trees within Wytham woods (image credit, imagery 2024 Airbus, Maxar technologies, map data 2024). Trees 3, 4, 5, 7, and 14 each contributed two tree holes for the experiment, the other trees contributed one. (**B)** Top inset—photograph of a control (left) and limed (right) tree hole. (**C)** Bottom inset—diagram of experimental design. Tree holes were monitored from 7 weeks before the start of the experimental perturbation. Limed tree holes then received a pulse of lime mix (CaCO_3_ and Na_2_CO_3_) at 0, 1, 2, 4, and 8 weeks after the onset of the treatment, with a final observation after 12 weeks. Week 0 sampling point was 2^nd^ July 2021, week 12 was 29^th^ September 2021. (**D)** The pH values over time in the control (black) and limed (green) tree holes, showing values before (solid lines) and after (dashed lines) the addition of lime for limed tree holes at each time point. Separate lines are shown for each tree hole. Asterisks show time points that lime was added to tree holes in the limed treatment.

We investigated ecological and evolutionary responses of decomposer communities of bacteria living in rainwater pools formed by the roots of beech trees, called tree holes, to perturbation (Fig. 1). It is one of the best studied environmental model systems of bacterial diversity, both in terms of field observation and experiments [30, 31] and from experimental evolution and functional assays in the laboratory [21, 32]. Nutrient inputs come primarily from leaf litter, especially during Autumn leaf fall. Previous work described the diversity of these communities sampled across South–East England and showed how diversity and composition influences several aspects of ecosystem functioning, such as decomposition rate, cellulose degradation, and phosphate metabolism [31, 32]. Previous studies on the evolution of tree hole bacteria in the lab found that increasing levels of diversity reduced the rate of adaptation of species to new abiotic conditions, namely low pH, although coevolution still led to enhanced ecosystem functioning [21]. Moreover, high diversity in tree hole communities constrained the evolution of focal species when exposed to environmental change in laboratory microcosms [22, 23]: the evolution of 22 focal strains grown factorially in eight different natural background communities depended almost equally on the strain, the background community, and strain by community interactions [22]. However, these experiments relied on simplified microcosms in the lab, and the findings might differ in realistically diverse bacterial communities living in ecosystems open to dispersal in the wild.

Here, we tracked the evolutionary and ecological dynamics of naturally occurring tree holes subjected to experimental perturbation by raising the pH with the addition of lime. Soil pH is the main abiotic determinant of variation in the composition of soil bacteria [33, 34], it is easy to manipulate by liming [35] and bacteria in the laboratory adapt to changes in pH over relatively short timescales [36]. A survey of soil bacterial community profiles across Great Britain provided strong evidence that pH structures bacterial taxa [37]. Higher pH soils (ranging up to pH >8) tended to support more diverse bacterial communities than acidic soils (down to pH <4), which supported bacterial communities dominated by a few specific families and phyla. Similar conclusions were reached by analysing soil bacterial genera globally, which vary widely in pH optima [38]. In laboratory experiments, pH causes ecological and evolutionary changes to communities over timescales of weeks where positive effects of diversity on community yields were intensified in the media with pH 5 compared to pH 7 [21]. Together, these studies justify the importance of pH for bacterial communities. Our goal is to quantify the relative importance of species sorting within communities, the arrival of new species by dispersal, and evolutionary genetic changes within species over a period of weeks and months when an important environmental parameter is manipulated [39]. We show that wild tree hole communities are robust to experimental perturbation by liming.

## Materials and methods

### Fieldwork and sampling

We collected samples from water filled tree holes during May 2021 to September 2021 from Oxford’s Wytham Woods field station (Fig. 1A, Table S1). Tree holes were chosen to be accessible and deep enough not to dry out during the experiment. Volumes were estimated based on the depth and diameter of tree holes assumed hemispherical shape. pH measured using a Lutron soil pH metre PH-212, calibrated on standard solutions. We initially intended pairing tree holes for control and liming treatment based on similar starting pH and volume, and selected tree holes for liming at random within such pairs, with some spares. Pairing was later dropped for analyses, however, as there was no natural basis in terms of 16S rRNA gene composition, and we present results for all tree holes including the spares. Treatment tree holes were limed in weeks 0 and 1 by adding 9:1 mix of CaCO_3_: Na_2_CO_3_ to attempt an estimated concentration of 1 g/litre (~0.01 M and 0.001 M of CaCO_3_ and Na_2_CO_3_, respectively). There was wide variation, however, in how much pH increased (possibly due to inaccurate volume estimates because of hidden extensions to tree holes or variation in the amount of buffering material such as leaf litter). Treatment tree holes therefore received variable amounts of lime mix aiming for an immediate increase in pH of >2 (Fig. 1D, amounts added to each tree hole recorded in Table S1). From week 2 onwards, we added just Na_2_CO_3_. In total, liming added 0.3 to 3.6 g/l of Ca + and 0.1 to 1.3 g/l of Na + ions as well as raising pH (Table S1). Control tree holes received no lime.

Sampling and liming treatment occurred on a partially expanding scale of 0, 1, 2, 4, 8, and 12 weeks to detect short- and long-term responses. After stirring to homogenize tree hole contents, 10 ml of liquid was collected per tree hole (prior to lime addition where applicable) per time point. A part of it was directly stored at −80°C for sequencing. The rest of the sample was used to inoculate 5 ml of sterile beech tea media that mimics natural resources for these species supplemented with Nystatin (1:100 dilution, Gibco) to suppress fungal growth. Stock beech tea media was prepared by autoclaving 50 g of dried beech leaves in 500 ml of deionized water, centrifugation, and filtration to remove coarse particles [32] then diluted 16-fold in sterile water for culturing. Cultures were incubated at 22°C in static conditions for 7 days and then stored at −80°C with a freezing solution of NaCl:glycerol (to a final concentration of 0.8% w/v: 30% v/v) as stock communities for phenotypic assays.

### 16S rRNA gene amplicon sequencing and analysis

Frozen liquid samples collected per tree hole were defrosted and up to 8 ml (less if lots of soil present) centrifuged for 5 min at 3220 g. DNA was extracted from the pellet using the QIAGEN Power Soil kit following the manufacturer’s standard protocol, with final elution in 50 μl DNA was submitted to Novogene for amplicon 250 bp paired end sequencing of the V3-V4 region of 16S rRNA gene on NovaSeq System (Illumina). Between 160 862 and 179 760 reads were sequenced per sample for weeks 0 and 8, and 81 001 to 98 908 reads for weeks 1,2, 4, and 12. More than 99% of bases had quality scores >20 in all samples. Novogene bioinformatics service ran reads through the QIIME2 pipeline to merge and filter reads to reduce noise and remove chimeras [40], assign reads to amplicon sequence variants (ASVs), calculate observed ASV count, and Shannon index statistics for each sample. We reran taxonomic identification by uploading the representative 16S rRNA gene sequence for each ASV to the RDP2 database online.

Read count data for each ASV were transformed to a centralized log ratio (CLR) in the R statistical programming language. Variation in compositional samples among treatments, sample times, and tree holes was analysed using the adonis2 function in the VEGAN package. Principal components analysis was conducted with the prcomp function and ellipses were plotted using the ordiellipse function in the VEGAN package. Linear mixed effects models were run with the CLR counts of each species as response variable in turn, treatment (control versus limed tree holes, where “limed” is only coded for samples from week 1 onwards, i.e. after the impact was applied) and sampling time as fixed effects and tree hole as a random effect, using the lme4, lmerTest, and rsq packages in R.

### Collection of focal isolates

We isolated focal bacteria both from environmental tree hole samples and from laboratory-grown stock communities derived from these samples. To assess the impact of lime treatment, we selected three independent replicates for both control and limed tree holes. Samples were taken at three time points: −7 weeks before liming started, representing baseline conditions; 0 weeks, collected immediately before liming started, again representing baseline; and 8 weeks, after liming started, reflecting the effects of lime treatment [22]. This constitutes a Before-After-Control-Impact design [41], with “Before” as pre-liming samples from weeks −7 and 0, “After” as post-liming samples in week 8, “Control” as the control tree holes that received no lime and “Impact” as the limed tree holes. Briefly, 50 μl of the tree hole sample was spread on R2A agar (Sigma-Aldrich, Gillingham, UK) and incubated at 22°C for 7 days. We then selected three colonies that displayed distinct morphological differences. These colonies were inoculated in 3 ml of beech tea media and incubated at 22°C. After four days, the resulting suspension was again spread onto R2A agar as a second round of purification and incubated for 3 days. The resulting single colonies were transferred to fresh beech tea media and incubated for 7 days to achieve maximum density. Bacteria in beech tea media grow slowly but for sustained periods (weeks to months) without media replacement [22], which likely minimizes rapid adaptation to lab conditions and loss of wild phenotypes. Final monoclonal isolates were stored at −80°C with a freezing solution of NaCl:glycerol (to a final concentration of 0.8% w/v: 30% v/v) before further use. For characterizing focal isolates, we extracted DNA from all the isolates using the DNeasy blood and tissue kit (Qiagen) following the manufacturer’s protocol. A 1465 bp fragment of the 16S rRNA gene was amplified using RedTaq Ready Mix (Sigma-Aldrich) and the primer set 27f/1492r (Sigma-Aldrich) for Sanger sequencing. PCR cycling parameters were as follows: 95°C for 5 min, followed by 30 cycles of 95°C for 30 s, 54°C for 30 s, and 72°C for 1.30 min, with a final extension time of 5 min at 72°C. Sequences were aligned in Geneious and identified using the RDP2 database online identification (Fig. S1) [42].

### Growth assays in a media with varying pH

All laboratory experiments were performed in 96 well microplates containing beech tea media and incubated at 22°C in static conditions unless stated otherwise. To assess the performance of control and lime-treated communities, as well as the focal isolates, we tracked their growth in beech tea media with varying pH levels. The pH was varied from 6.0 (original beech tea media pH) to 7.5 and 8.2 using a 9:1 lime mix of CaCO_3_: Na_2_CO_3_ prepared by adding 0.01 M and 0.001 M of CaCO_3_ and Na_2_CO_3_, respectively at the start of the experiment to mimic the pulse lime addition in the wild. We did not buffer the media to maintain a constant pH. Entire communities and focal isolates from three time points: −7 weeks, 0 weeks, and 8 weeks, were first grown in beech tea media at pH 6.0 for 7 days, then inoculated into fresh media with pH 6.0, 7.5, and 8.2 at a 1:100 dilution, and incubated again at 22°C for 7 days. We evaluated the growth of the communities and the isolates by measuring the optical density (OD) each day and calculating the area under the curve (AUC) using the Growthcurver package in R.

### Modified time shift assay

Time-shift assays are typically used to measure coevolutionary dynamics between pairs of species over multiple time points in an evolution experiment [20, 43]. In our study, we employed a modified time-shift assay to investigate microbial community interactions in tree hole ecosystems. Briefly, communities from three time points, −7 weeks, 0 weeks, and 8 weeks for each tree hole were initially grown in beech tea media for seven days. After incubation, we filter-sterilized the cultures using 0.45-micron filters to produce spent media for subsequent assays (Fig. 7A). Following a standard time-shift scheme, we then inoculated fresh community inocula from the “week 0” community for each tree hole on spent media derived from the same “week 0” community, spent media from the “week −7” community (i.e. the past), and spent media from the “week 8” community (i.e. the future), all originating from the same tree hole. If community composition changed over time in tree holes in relation to resource use, bioactive molecule production, or other properties that affect species interactions, we expected growth on spent media to vary depending on which spent media is used. For a fully factorial experiment, we also grew “week −7” and “week 8” community inocula on all three versions of spent media (resulting in a total of 3 × 3 combinations per tree hole). We monitored the growth of the community × spent media combinations by measuring OD daily from Day 0 to Day 7 and calculated the area under the curve (AUC) for growth over time. Interaction coefficients for each combination were estimated as described in the Results, and we analyzed the variation using linear mixed-effects models to assess the influence of time point and community interactions, and whether patterns differed between control and limed tree holes.

### Metagenome sequencing and assembly

We extracted DNA from all tree hole samples at two timepoints (week 0 and week 8) using the same procedures as described above for 16S rRNA gene amplicon sequencing. Samples were sequenced using Illumina (250 bp-paired-end) shotgun sequencing at Novogene. Cutadapt was used to remove adapters and trimmomatic was used to remove low-quality reads. Metagenome assembled genomes (MAGs) were constructed from high-quality paired reads using metaSPAdes [44]. The resulting scaffolds were clustered into bins representing the same genome at both timepoints using MaxBin (v2.0) [45], producing 770 binned MAGs representing putative species (see assembly statistics in Table S6) [46]. We used default settings in all analyses unless specified.

We assessed the quality of the assembled MAGs using CheckM [47] and filtered out low-quality bins. We retained 202 medium-quality MAGs with contamination <10% and completeness >50%. We clustered these MAGs at a 95% similarity threshold using the Mash and fastANI algorithms implemented in dRep [48]. We found some clusters of MAGs from different tree holes (i.e. we captured the same species from multiple locations), but no instances of MAGs from the same tree hole occurring within the same cluster (see Table S8). We are therefore confident that, within a given tree hole, each MAGs assembled represents a different species, rather than different strains of the same species. We annotated these MAGs using Prokka (v1.14.5, [49]) to identify genome features in the sequences. We then estimated the in-situ growth rates of these metagenome assemblies at both timepoints using Compute PTR (CoPTR). Growing species will have higher sequencing coverage at the origin of replication as DNA is sequentially replicated from the origin to the terminus. CoPTR estimates the peak-to-trough ratio (PTR); the ratio of sequencing coverage near the origin of replication compared to the replication terminus. The log_2_(PTR) has been shown to correlate with bacterial growth rates [42].

### Variant calling

We selected the 22 MAGs with abundance (coverage) ≥10 at both timepoints to investigate sequence variants. Due to their high coverage, these MAGs are of much higher quality than the general quality threshold set to retain MAGS for analysis (completeness all >80%, contamination all <5%, see Table S9). We classified the taxonomy of these MAGs against the Genome Taxonomy Database using GTDB-Tk (v2.3.2) [50]. Quality and taxonomic information for these MAGs are detailed in Table S9. We used bbmap to map the original sequencing reads back onto the assembled MAGs and then used the callvariants.sh script implemented within bbmap to identify sequence variants [51]. We filtered these to a high quality single nucleotide variants (SNVs; ≥35 mapping quality) occurring on scaffolds that we are confident are well assembled (≥10 000 bp length). We then used the sequence annotations to identify SNVs occurring in gene-coding regions, and whether they were synonymous or non-synonymous, i.e. confer a change in the resulting amino acid sequence.

### Estimation of effective population sizes and generation times

We calculated Watterson’s *θ* per site as *K/a_n_*/*L* for each MAG in turn where *K* is the number of SNVs at the first time-point, *a_n_* is the (*n*-1)^th^ harmonic number, *n* is the average read depth of the MAG and *L* is the total length of the MAG in nucleotides. We then estimated *N_e_* for each MAG as *θ*/(2 *m*), where *m* is the median of mutation rates per nucleotide estimated for 16 bacterial species reported in Bobay and Ochman, 2018 [52]. We used medians because of skews to high values. To estimate the expected number of generations between sampling points for each MAG, we first calculated the standardized variance in SNV frequencies as *F* = (*x*-*y*)^2^/{*x*(1-*x*)}, for each SNV, where *x* is the frequency at time point 1 and *y* is the frequency at time point 2 [53]. Then the expected number of generations, *t* is - 2*N_e_*ln(1-*F*) and generation time in days is 56/*t*, where 56 days elapsed between sampling points. See Supplementary Text S1 for further details.

## Results

### Short-term and longer-term effects of liming on pH

We exposed eight tree holes to a pulsed liming treatment and compared their responses to nine control tree holes (Fig. 1, Table S1). Tree holes were sampled periodically before and after the start of the liming treatment, with pulses delivered to treatment tree holes at every sampling point from 0 to 12 weeks (Fig. 1C). Liming led to major short-term increases in pH of on average 1.6 +/− 0.19 (95% confidence interval) units after addition (shown by dashed green lines from weeks 0 onwards on Fig. 1D, black lines are control tree holes). A more moderate but persistent increase of 0.76 +/− 0.44 units (95% CI, solid green lines Fig. 1D) was measured before each pulse relative to control tree holes by the end of the liming treatment (comparing solid green lines to the black lines on the final day: t = 3.78, df = 12, *P* = .003, Fig. 1D).

### Community diversity increased marginally in limed tree holes

**Figure 2:**
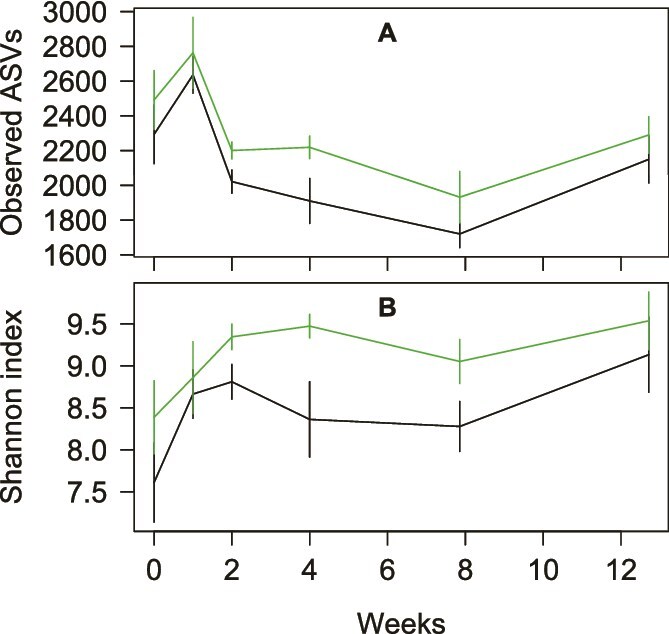
**(A)** Observed number of 16S rRNA gene amplicon sequence variants (ASVs) per tree hole over time. **(B)** Shannon indices of ASVs over time, black = control tree holes, green = limed tree holes. Standard errors are shown.

DNA was extracted from liquid samples collected over 0 to 12 weeks from control and treatment tree holes for 16S rRNA gene sequencing. Tree hole communities contained between 1127 and 3325 ASVs per tree hole, which represented between 542 and 984 genera based on matches to the RDP11 database (Table S2). Relative abundances were uneven, with e.g. 1.8 to 45.5% of all 16S rRNA gene reads belonging to the most abundant genus in each sample. There was no significant change in richness over time or between treatments, measured as observed numbers of ASVs (mixed effects model with tree hole identity as a random effect, and treatment, *F_1,36_* = 0.02, *P* = .89, and day *F_1,86_* = 2.4, *P* = .12 as fixed effects, Fig. 2A). Shannon diversity did increase significantly in limed tree holes (average increase 0.68+/−0.26 S.E, *F*_1,62_ = 6.7, *P* = .011), indicating reduced dominance of the most frequent taxa and greater evenness in abundance across taxa (Fig. 2B). Shannon diversity also increased significantly over time across all the tree holes, independently of treatment (slope = 0.045 per week, *F*_1,89_ = 5.1, *P* = .027). The difference between limed and control tree holes was greatest from week 1 to week 4 and decreased after 8 weeks (standard errors overlap at later times, Fig. 2B).

### Community composition also shifted significantly but marginally in limed tree holes

**Figure 3:**
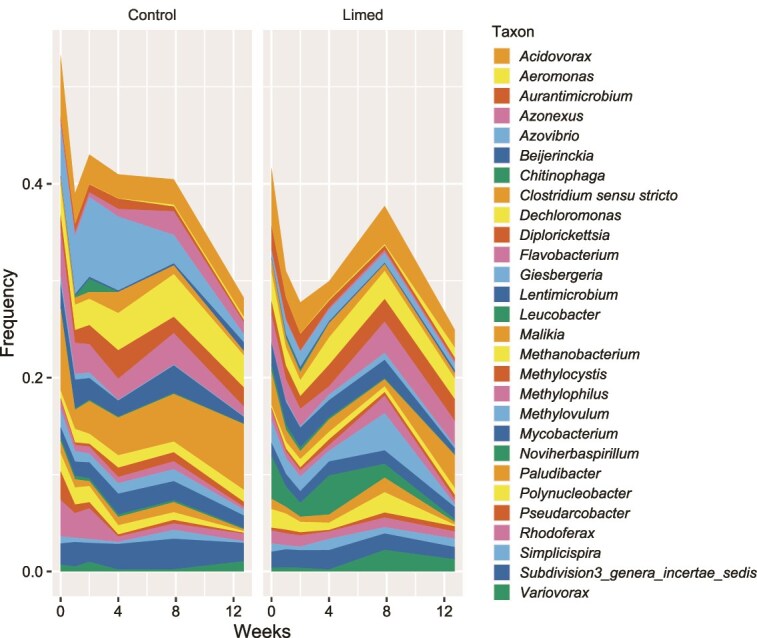
The frequencies of 28 genera that had a frequency of at least 5% in any single sample. Left panel—average across control tree holes, and right panel—average across limed tree holes. Colours recycle through a colour-blind palette, hence each used four times, but in the same order on the plots as in the legend.

**Figure 4:**
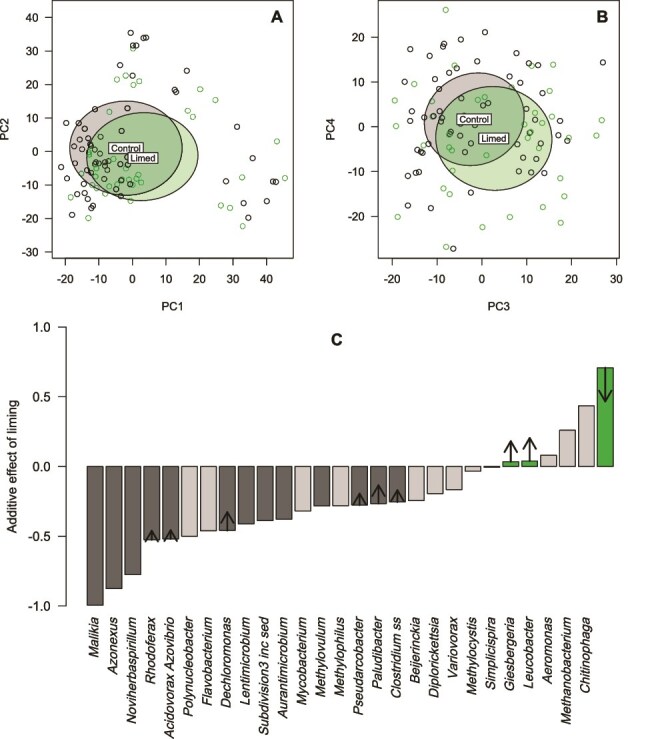
The effects of the liming treatment on the taxonomic composition of tree holes. Top row—principal components analysis of centred log ratio transformed read counts of genera, showing standard deviation ellipses around the centroid for each factor. (**A**) PC2 versus PC1, (**B**) PC4 versus PC3. PC1 to PC4 explained 18.5% of the variation in tree hole composition among samples. All tree holes prior to the start of the liming treatment were classified as “control”. Bottom row—(**C)** the additive effect size of liming treatment on the centred log ratio counts in linear mixed effect models fitted to each genus in turn with fixed effects of treatment and time, and tree hole as a random effect. Arrows—magnitude and direction of significant interaction terms between treatment and time. Dark grey bars indicate significant decrease in limed tree holes. Green bars indicate significant increase in limed tree holes.

Composition varied among tree holes and showed turnover in the most abundant taxa over time in both control and limed tree holes (shown for 28 genera with a frequency > 0.05 in any sample, Fig. 3, Fig. S2). We used the natural log centralized ratio transformation of read counts as the response variable for statistical analysis of variation in taxonomic composition [54]. Overall, there were significant additive effects of liming treatment, time, and tree hole in explaining compositional variation in CLR counts among samples (Adonis: Liming treatment, *R*^2^ = 0.01, *P* < .001; Week, *R*^2^ = 0.04, *P* < .001; Tree hole, *R*^2^ = 0.22, *P* < .001, Fig. 4A and B, Fig. S3), in ascending order of importance. Although significant, the liming treatment has a relatively small effect, and the distribution of compositions between the control and limed tree holes were displaced but broadly overlapping (Fig. 4A and B).

To identify which genera were most impacted by liming, we ran separate linear mixed effects models with liming treatment and week as fixed effects and tree hole as a random effect for each genus in turn. Out of 1998 genera with frequency of at least 0.001 in any sample, 350 (17.5%, Table S3) displayed a significant effect of liming treatment on their CLR count in separate linear models (significantly more than expected from repeated tests with false positive rate α = 0.05, binomial test *P* < .00001). The effects were either additive or in interaction with the sampling week (for comparison, 775 showed a significant effect of time). Out of the 28 genera with frequency > 0.05 in any sample, 14 showed a significant effect of liming (Fig. 4C, Fig. S4, again a higher proportion than expected from false positives due to multiple tests, binomial test *P* < .00001): 13 were alkaliphobes that decreased in relative frequency (dark grey bars, Fig. 4C) and three were alkaliphiles that increased in relative frequency (green bars, Fig. 4C). Nine genera showed significant interactive effects of treatment and time (arrows, Fig. 4C). In nearly every case this represented a relative increase in abundance in limed tree holes at later time points, except for the genus (*Chitinophaga*) that increased most in limed tree holes, but less so at later time-points.

### Colonisation by new taxa had a relatively small effect on tree hole community composition

**Figure 5:**
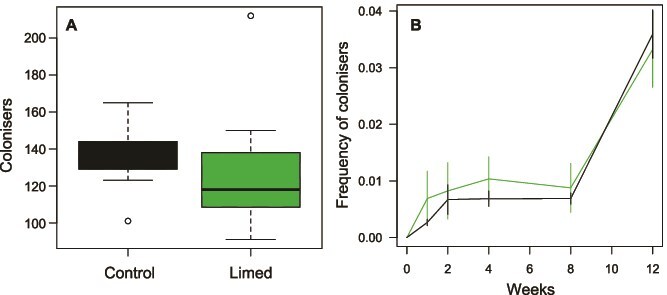
The number (**A**) and frequency (**B**) of genera that “colonised” the tree holes during the experiments based on 16S rRNA gene sequences. Colonisers were defined as genera that appeared during the experiment, hence absent at week 0, and once appeared remained present until the end. In (A) the box shows the interquartile range, the bar shows the median, and the whiskers show the most extreme data point no more than 1.5 times the interquartile range. In (B), the frequency was calculated per community sample as the number of 16S rRNA gene reads identified as dispersers divided by the total number of 16S rRNA gene reads, then averaged across control (black) and tree hole (green) communities.

We quantified the number of genera that appeared in later samples of a given tree hole during the post-liming period that were not present before liming started and remained present after their first observation. On average 133.1 ± 7.0 genera appeared per tree hole by the end of the experiment, with no significant difference in number between limed tree holes and control tree holes (t-test t = 0.52, df = 10.3, *P* = .62, Fig. 5A). These “colonisers” constituted on average 3.5 ± 0.3% of the community by week 12, consistent with an average colonisation rate into the community of 0.2% constituting on average 11 new genera per week. The average frequency of “colonisers” increased over time, but there was no significant effect of the liming treatment (mixed effects model with tree hole identity as a random effect, and treatment, *F_1,17_* = 0.1, *P* = .71, and week *F_1,68_* = 111.1, *P* < .0001, as fixed effects, Fig. 5B). ASVs show a similar pattern of no significant difference between treatments (Fig. S5), but with higher rates of apparent colonisation than genera (to be expected due to their nesting within genera): 2.1% frequency constituting 177 ASVs per week. “Colonisers” could represent growth from lower densities than detectable with our depth of coverage from 16S rRNA gene sequencing, growth of new taxa from dormant stages such as spores, colonisation from taxa attached to tree hole walls, or true invasion from outside the tree hole. For this reason, we consider it an upper estimate for the effect of dispersal on changes in community composition.

We quantified the degree of connectivity among tree holes based on 16S rRNA gene ASV sharing, as an indicator of longer-term dispersal. On average, sharing was relatively high, with 34% of reads matching to identical ASVs between any pair of tree holes (ranging from 16% to 57%), indicating high connectivity. There was no significant effect of geographical distance between tree holes on the degree of sharing (mantel permutation test, r = 0.04, *P* = .33, Fig. S6): some pairs of neighbouring tree holes had relatively high ASV sharing (e.g. 14 L and 14R, 53%) but other neighbouring tree holes were less similar (e.g. tree hole 7A and 7B, 25%, Fig. S6).

### Growth of communities and focal isolates remained unaffected despite liming treatment

**Figure 6:**
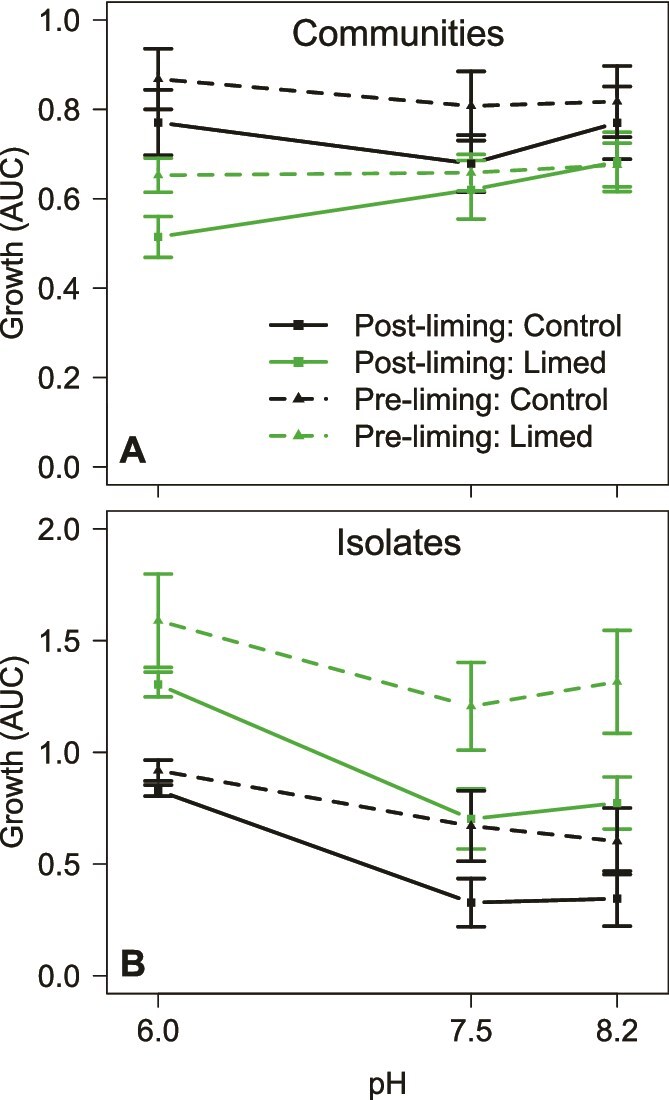
Mean growth of tree hole communities (**A**) and isolates (**B**) in the laboratory on beech tea medium manipulated to different pH values. Growth was measured as the area under the curve. Dashed lines are for communities sampled before the liming treatment started (pre-liming = weeks −7 and 0 combined), solid lines are for communities sampled after the liming treatment started (post-liming = week 8). Black—average and standard errors for the control tree holes, green—limed treatment tree holes.

To test whether bacterial communities adapted to liming, we grew entire community samples on beech tea medium with a pH ranging from 6.0 (the default pH of beech tea medium) to 8.2 in the laboratory. The growth of entire communities was not affected significantly by the pH of the beech tea media (Fig. 6A, Table S4). There was a weak trend for communities collected from limed tree holes after the treatment started (post-liming) to grow better at pH 8.2 than at lower pH values, but this was not significant. Sampling time was marginally significant: samples from after the treatment grew better than samples of the corresponding tree hole before, but in both control and limed tree holes, hence due to an uncontrolled factor affecting all tree holes rather than our treatment (Table S4). Similarly to the observation of pH buffering in wild tree holes, cultured bacteria brought media pH down to 5.5 or 6.0 after 7 days of growth in all experimental treatments, which is evidence of resilience to the experimental perturbation.

We grew a set of focal isolates from the same time points (sampled from three tree holes of each treatment, Materials and methods) on beech tea media with varying pH. Focal isolates showed significant additive effects of pH, treatments and timing of the sample (Fig. 6B): isolates grew better on average at pH 6.0, isolates from limed tree holes grew better than those from control tree holes, and isolates from the pre-lime time periods grew better than those from post-lime times (Table S4). There was no evidence, however, for significant interactions, which would be expected if isolates from tree holes experiencing liming grew significantly better at higher pH, and reduced growth at the later time point was observed in control tree holes as well, hence not attributable to liming treatment per se.

### Time shift assay indicates variation in whole community phenotypes

**Figure 7:**
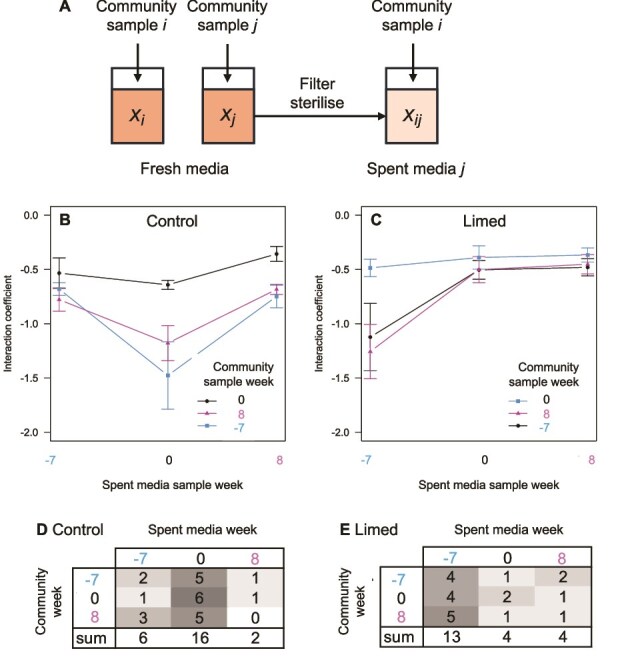
Time shift assay of interaction coefficients between whole communities isolated from different times during the experiment estimated through growth on spent media. Community inocula were sampled from week −7, week 0, and week 8. (**A**) In a single assay, community samples from two time points, *i* and *j*, from the same tree hole were grown on fresh beech tea medium and growth recorded as *x_i_* and *x_j_*, respectively. Community sample *i* was then grown on spent media filter sterilized from community *j* and growth was recorded as *x_ij_*. The interaction coefficient = (*x_ij_* - *x_i_*)/*x_j_*. (**B**) The mean interaction coefficient across control tree hole community assays. X-axis indicates the time point of the community used to generate spent media, *j*, separate lines the time point of community grown on that media, *i*. All means are negative indicating that growth is lower on spent media than on fresh media. (**C**) The mean interaction coefficient across community assays for tree holes in the lime treatments. (**D**) Tally of the number of cases across control tree holes of which spent media, *j* (columns), reduced growth of community sample *i* (row) most (shaded proportional to count to highlight variation). (**E**) The same tally for limed tree holes. An excess of cases in the diagonal of the table would indicate a tendency for self-inhibition. Instead, spent media from week 0 for control tree holes and week −7 for limed tree holes have a uniform greater reduction on growth across all community types.

To determine the proportion of evolution driven by coevolution with other species (biotic factors), we performed a time shift assay [55]. The goal is to assess community-wide phenotypic changes over time by comparing the growth of entire microbial communities, as well as focal isolates, from contemporary conditions to those from earlier and later time points within the same tree hole [20, 56]. Each assay grew community samples from two time points, *i* and *j*, from the same tree hole on fresh beech tea medium and recorded growth as the area under the curve, *x_i_* and *x_j_*, respectively (Fig. 7A). The culture from community sample *j* was then filter sterilized to produce spent media *j*. Community sample *i* was added to the spent media *j* and its growth was recorded as *x_ij_* (Fig. 7A). We then calculated an interaction coefficient as (*x_ij_* - *x_i_*)/*x_j_*, which represents the effect of community *j* on community *i*, scaled by how well community *j* grew on the fresh medium (see Supplementary text S1 for justification). A negative value indicates that growth is inhibited on spent media (e.g. because shared resources have been used up), whereas a positive value indicates stimulation by spent media (e.g. community *j* might detoxify compounds in the medium such as tannins or release useful metabolites from recalcitrant substrates [32]). Assays were repeated for time *i* and *j* for all factorial combinations of −7 weeks (reflecting the “past”), 0 weeks (the “present”) and 8 weeks (the “future”) relative to the start of the liming treatment. If communities remained constant in their resource use and production of inhibitory or stimulatory biomolecules over time, we expect the same interaction coefficient irrespective of spent media type (i.e. for all *j*). If such factors vary over time, however, interaction coefficients should vary. Specifically, we might expect a difference between self (*i* = *j*) versus other comparisons (*i*  $\ne$*j*).

Interaction coefficients were generally negative, as communities grew worse on spent media than on fresh media (Fig. 7B, C). There was significant variation in interaction coefficient across assays (Table S5), however, primarily with respect to the sampling time *j* of the community used to generate the spent media. On average, spent media from week 0 for control tree holes and spent media from week −7 for limed tree holes caused the strongest reduction in growth (Fig. 7B, C: control tree holes effect of spent media, ANOVA *F_2,204_* = 12.3, *P* < .0001; limed tree holes effect of spent media, ANOVA *F_2,154_* = 9.8, *P* < .0001, Table S5). Different tree holes showed different patterns (fixed effects *R*^2^ = 0.15, random effects *R*^2^ = 0.16, residual *R*^2^ = 0.69), with some displaying the greatest growth reduction with same time comparisons (*i* = *j*, counts in table diagonals in Fig. 7D, E), whereas other tree holes showed the greatest reduction with different time comparisons (*i*  $\ne$*j*, off diagonals in Fig. 7D, E). The same average tendency was observed, however, that spent media from week 0 for control tree holes (16/24 tree holes, multinomial test of column sums, *P* = .002) and from week −7 for limed tree holes (13/21 tree holes, multinomial test, *P* = .032) were more likely to yield the greatest growth reduction irrespective of focal community *i*. The observed differences cannot easily be ascribed to a causal effect of liming, however, as the week 0, samples had not yet been exposed to liming either: any variation directly attributable to liming should lead to a distinction between week 8 samples versus weeks −7 and 0.

### Evolutionary responses of metagenome assembled genomes

To investigate genetic change over time, we constructed metagenome assembled genomes (MAGs) from shotgun sequences at week 0 (pre-lime) and week 8 (post-lime), which represent putative species. By mapping the raw sequencing reads back to the assemblies, we were able to quantify the frequencies of SNVs at the two timepoints in the 22 most abundant MAGs (taxon identifications in Fig. S7). Within these we identified SNVs as synonymous (s-SNVs; not causing a change in amino-acid sequence) or non-synonymous (ns-SNVs; causing an amino-acid change). Among these ns-SNVs, 42 (6.9%) were new (occurred at the second timepoint, but not the first), of which 7 were considered “sweeping” variants (frequency changed above 75% at the second timepoint; supplementary Fig. S7). These new sweeping variants were all found on the same MAG, a members of the order *Solirubrobacterales* from the limed tree hole 14R. One of the sweeping variants occurred on a Heme A synthase gene, however, the others all occurred on genes annotated as hypothetical proteins.

### Intraspecific and interspecific responses are of equivalent magnitude and inter-linked

**Figure 8:**
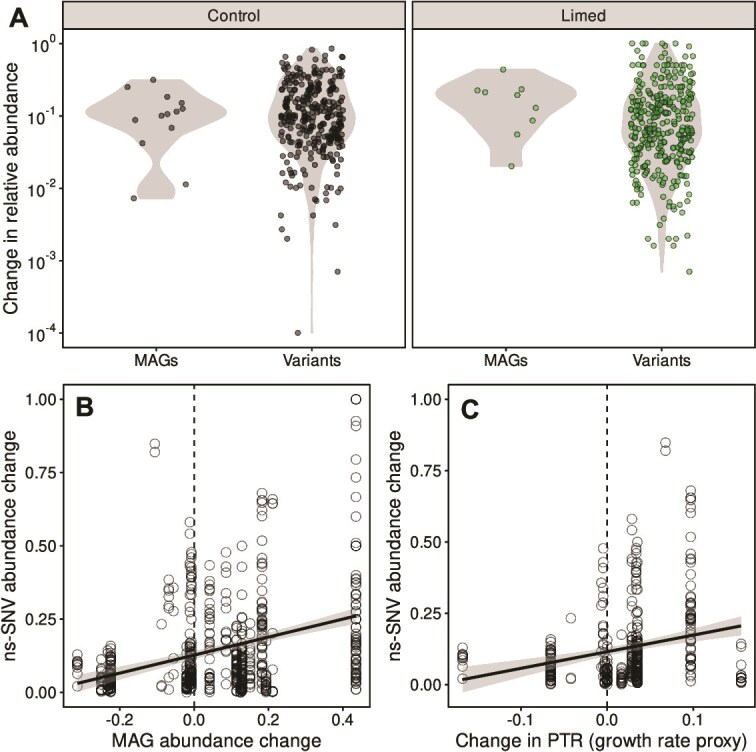
Inter- vs intra-specific sorting. (**A**) The distributions of absolute changes in relative abundance of MAGs and non-synonymous single nucleotide variants (ns-SNVs) are shown as violin plots with all data points overlaid, for control (black) and limed (green) tree holes respectively. Points are shown in log_10_ scale due to their highly right-skewed distributions. We found no significant difference between the changes in abundance of MAGs and ns-SNVs in either control (Wilcoxon rank sum test, *P* = .84) or limed tree holes (*P* = .12). (**B**) Changes in ns-SNV abundance are strongly linked to changes in the abundance of the MAGs they are found on (linear regression, intercept = 0.13, slope = 0.31, *R*^2^ = 0.12, *P* < .001). (**C**) Changes in ns-SNV abundance are also linked to the PTR, a proxy for in-situ growth rate (linear regression, intercept = 0.12, slope = 0.58, *R*^2^ = 0.06, *P* < .001). Vertical dashed lines highlight zero in plots B and C below which relative abundance or PTR respectively are reduced between the timepoints, and vice versa. There are fewer data points for PTR (C) than changes in MAG abundance (B), as PTR could not be estimated for all MAGs at each timepoint.

We considered changes in the frequencies of ns-SNVs as intra-specific sorting (i.e. evolution) and changes in the abundances of the MAGs as inter-specific sorting (i.e. ecological). We compared the distributions of changes in the relative abundance of the MAGs to changes in frequencies of the ns-SNVs (Fig. 8A and B) and found no significant differences for MAGs from either control or limed tree holes (Wilcoxon rank sum tests; control *P* = .84, limed *P* = .12). This analysis quantified the magnitude of change in MAG abundance (not separating increases from decreases) as most appropriate to compare to the changes in SNV frequency, which cannot be considered directional. We also asked whether species that increased in frequency over time (i.e. ecological winners) also showed greater changes in frequencies of variants (i.e. more evidence of evolution). We quantified the signed changes in MAG relative abundances, i.e. accounting for both increases and decreases between timepoints. We found that MAGs that increased in relative abundance had greater changes in ns-SNV frequencies (linear regression, intercept = 0.13, slope = 0.31, *P* < .001, Fig. 8B) even after accounting for differences between tree holes. However, we found no effect of the liming treatment (mixed effects model with tree hole identity as a random effect, and MAG abundance change, *F_1,191_* = 11.8, *P* < .001, and treatment *F_1,7_* = 0.08, *P* = .78, as fixed effects). We also tested whether this response was consistent between communities by regressing the changes in SNV frequency against the changes in MAG relative abundance in each tree hole represented by >1 MAG. Although most of these responses were non-significant due to limited numbers of MAGs available per community, five out of six tree holes showed a positive slope (linear regression), demonstrating consistency in response across tree hole communities (Fig. S8).

The increased relative abundance of some of the MAGs may be due to increased growth rates of those species. Growth rates can be estimated from metagenome data using the peak-to-trough ratio (PTR), which is the ratio of coverage near the replication origin to the replication terminus of the MAG and has been shown to correlate with bacterial growth rates [57]. For each MAG, we calculated the PTR as a proxy for the growth rate at each timepoint, and investigated whether increases or decreases in growth rate were linked to changes in ns-SNV frequencies (Fig. 8C). We found MAGs which increased in growth rate between the two timepoints had greater changes in ns-SNV frequencies (linear regression, intercept = 0.12, slope = 0.58, *P* < .001, Fig. 8C). The effect remains after accounting for differences between tree holes (mixed effects model with tree hole identity as a random effect, and PTR change, *F_1,117_* = 5.4, *P* = .021, and treatment *F_1,4_* = 8.3, *P* = .048, as fixed effects). We see a small effect of the treatment; however, this is a reduced dataset compared to the changes in MAG abundance as PTR could not be calculated for all MAGs at both timepoints.

### Calibration of MAG polymorphism and allele frequency changes

We used rough calculations to evaluate our observations of MAG evolution. First, based on observed SNV polymorphism we estimated a median Watterson’s *θ* per base as 4.5 × 10^−6^ (95% quantiles 2.2 × 10^−7^ to 3.7 × 10^−5^ across MAGs). Assuming a mutation rate of 2.0 × 10^−10^ per nucleotide per generation (the median of estimates from sixteen bacteria reported by Bobay and Ochman [52]), this yields a median effective population size, *N_e_*, of 10 895 (95% quantiles 528–89 845 across MAGs, Table S7). This estimate seems low compared to estimates from global samples of sequence variation [36] and is lower than the likely census population sizes in each tree hole. We then estimated the number of generations that elapsed between sampling points based on the expression for the expected variance in SNV frequency changes at silent sites under drift from [53]. We detected a median of 6.6% changes in the frequency of silent SNVs per SNV per MAG over the 56 days between metagenome samples (Table S7). Combined with the *N_e_* estimates, this yields an average estimated generation time of 29.3 hours (95% quantiles 1.7 hours to 1161 days), which is longer than the minimum generation times in rich conditions in the laboratory. This outcome seems plausible for low nutrient conditions. For instance, a previous study [23] estimated a generation time of ~28.8 hours (70 generations in 12 weeks) for four bacterial species growing on beech tea in the laboratory.

## Discussion

Our study quantifies the tempo of eco-evolutionary change in wild bacterial communities in response to an experimental perturbation to see how quickly and in what way the whole system responds. We applied liming as an environmental perturbation to wild tree holes, based on the general importance of pH in influencing bacterial adaptation and variation in bacterial communities [34, 58–60]. Overall, tree hole bacterial communities were robust in the face of the manipulation and appeared to “buffer” the changes. The initial jump to high pH (>pH 8 to 10) returned relatively quickly to lower pH each time lime was added. Bacteria can modify their environment by changing pH and the metabolism of decomposer communities is generally acidifying [61]. Yet, repeated pulses led to a persistent increase of almost 1 pH unit on average by later time points, which is large enough to be expected to elicit physiological effects [62] and changes in the community composition in soil [35, 37]. Nonetheless, responses by the tree hole communities were small. Other perturbations, such as acidifying the communities, might have generated larger community responses.

Breaking down community responses into ecological species sorting, dispersal, and within-species evolution (i.e. changes in SNV frequency within MAGs), we found a treatment effect on species sorting, but not on dispersal or evolution. Similar results have been observed for polluted lotic ecosystems at micro-geographical scales where species sorting is dominant over dispersal [63]. Community-level assays confirmed functional redundancy among communities, with no consistent variation in the ability to grow on different pH conditions. Communities did vary in joint phenotypic characteristics detected by growth on spent media, which could reflect changes in either resource use and/or production of bioactive molecules [22]. None of these differences could be attributed to a causal effect of liming. Tree hole communities therefore seem quite resilient to pH change, suggesting that maybe communities are not so sensitive in the face of this environmental stress (despite evidence for large responses of bacterial composition to similar pH changes at geographical scales [37]). This also holds true for host-associated microbial communities as evidenced by a recent study which shows how ecological barriers restrict colonization in adult gut microbiome even after strong antibiotic perturbation [64].

One question regarding community responses is whether local communities such as tree holes represent isolated communities versus simply samples of a wider community connected by high rates of dispersal, and whether dispersal promotes or inhibits response to perturbations [11, 65–68]. We did detect evidence of colonisation via dispersal and evolution. However, the magnitude of those processes was the same in limed and control tree holes. Most of the change in taxon relative frequencies was due to sorting found in the tree hole initially rather than colonisation via dispersal (which represented <3% of frequency changes). Tree holes are generally well connected with high overlap in ASVs [30], consistent with relatively high rates of invasion and dispersal either from a common pool or between them over longer periods. In every putative case of colonisation by dispersal here, the same ASV was observed in at least one other tree hole in our sample. Yet, over the time scale of 12 weeks, it contributed a small fraction of changes in frequency.

Our approach to estimating dispersal based on ASV sharing provides a coarse-grained measure of connectivity among tree holes but does not resolve strain-level differences within shared ASVs. It is possible that some of the ASVs identified as shared between tree holes represent distinct, independently acquired bacterial strains rather than true dispersal events. Recent work has highlighted how shared environments can lead to the overestimation of microbial transmission when relying on species- or ASV-level resolution [69]. Hence, our results indicate an upper estimate of dispersal based on high connectivity among tree holes at the ASV level, rather than definitive evidence of transmission. Future work using strain-resolved amplicon sequencing or genomic variation within ASVs could provide a more precise assessment of dispersal and colonisation dynamics.

Evolution was detected as genetic changes in SNV frequencies in the MAGs. In high diversity communities (several thousand ASVs based on 16S rRNA gene sequencing) it is challenging to match up earlier and later samples and be sure of comparing the same genome, but coverage was high enough to track 22 high frequency MAGs over time. We detected a median of 7.6% and 6.6% changes in the frequency of coding and silent SNVs, respectively, per MAG per week, but no difference in rate between limed and control tree holes. Very rough calculations comparing levels of polymorphism with the frequency of silent SNV changes (and literature estimates of mutation rate [70, 71]) support generation times of around 1 per day that correspond to the previous estimates of growth rates on beech tea in the laboratory [23], hence around 46 generations (median across MAGs) elapsed within 8 weeks. Effective population sizes were low at around 10^4^ compared to estimates of 10^8^ or so in literature compilations based on the more global sampling of bacterial species [52], reflecting smaller isolated populations of bacteria within each tree holes, or a history of recent colonization and/or turnover of genotypes within them. Those MAGs that increased in relative abundance by the later time-point were those with greater average changes in coding SNV frequency, which is consistent with the increase in relative population size being associated with the spread of a new genotype. PTR results reflected a similar pattern – those species that increased in growth rate also showed the most sorting of within-species genotypes. Furthermore, the magnitude of ecological sorting and evolution were equivalent in terms of % changes in relative frequency, consistent with similar magnitudes of intra- and inter-specific sorting as evident for siderophore evolution [6]. Changes over time within MAGs could encompass both the replacement of one strain by another (providing they share >95% identity as defined by our cut-offs), and the accumulation of genetic changes within a single strain or interacting population of bacteria. Although the distinction between species and strains is hard to define conceptually, by adopting standardized genomic similarity metrics that reflect a pragmatic species level, we can partition variation consistently among taxa and tree holes.

Most evolution experiments have focused on laboratory populations [72, 73], and for good reasons because of the degree of control of conditions and ease of measuring outcomes (especially in monocultures, extensively reviewed in [73]). Our experiment occupies the opposite end of the spectrum where we tested whether the change in a single condition commonly studied in laboratories is sufficient to cause dramatic effects in wild bacterial communities. Much of the variation we measured was observed for specific sampling times or between different tree holes, which potentially reflect additional changes in conditions. For instance, the final sampling time coincided with the onset of autumn leaf fall, which is expected to change nutrient availability in the tree holes.

The lack of response despite a relatively large perturbation of 1–2 pH units provides evidence for the resilience of bacterial communities to short term perturbations in their pH environment. It is an important result because it addresses a crucial knowledge gap about the resistance and recovery of microbial communities to environmental perturbations [74]. Being resilient to perturbations allows microbial communities to maintain essential ecosystem functions, resist environmental fluctuations, protect host organisms, and adapt through evolutionary mechanisms [39, 75, 76]. These strategies ensure that ecosystems remain stable, productive, and functional even under challenging environmental conditions. Hence, the approach of experimentally manipulating wild communities and tracking multiple responses could be usefully applied to a broad range of microbial communities to improve understanding of how they respond to an environmental change.

## Supplementary Material

SupplementaryFigure1_final_wraf144

SupplementaryFigure2_final_wraf144

SupplementaryFigure3_final_wraf144

SupplementaryFigure4_final_wraf144

SupplementaryFigure5_final_wraf144

SupplementaryFigure6_final_wraf144

SupplementaryFigure7_final_wraf144

SupplementaryFigure8_final_wraf144

Supplementary_table_final_wraf144

Supplementary_information_wraf144

## Data Availability

DNA sequence data are deposited in BioProject PRJNA1223688 in Genbank: 16S rRNA gene sequencing data have accessions SAMN46824442–SAMN46824543 and MAGs have accessions SAMN47603880–SAMN47603901. Metagenome shotgun sequencing reads are on the short read archive with accessions SRR32774235–SRR32774268.
