# Supplementary material for: Eco-evolutionary robustness of wild bacterial communities to experimental perturbation
Source: ISME J. 2025 Jul 22;19(1):wraf144. doi: 10.1093/ismejo/wraf144 (PMC12743297; doi:10.1093/ismejo/wraf144)
Supplement: SupplementaryFigure1_final_wraf144 [file supplementaryfigure1_final_wraf144.pdf]

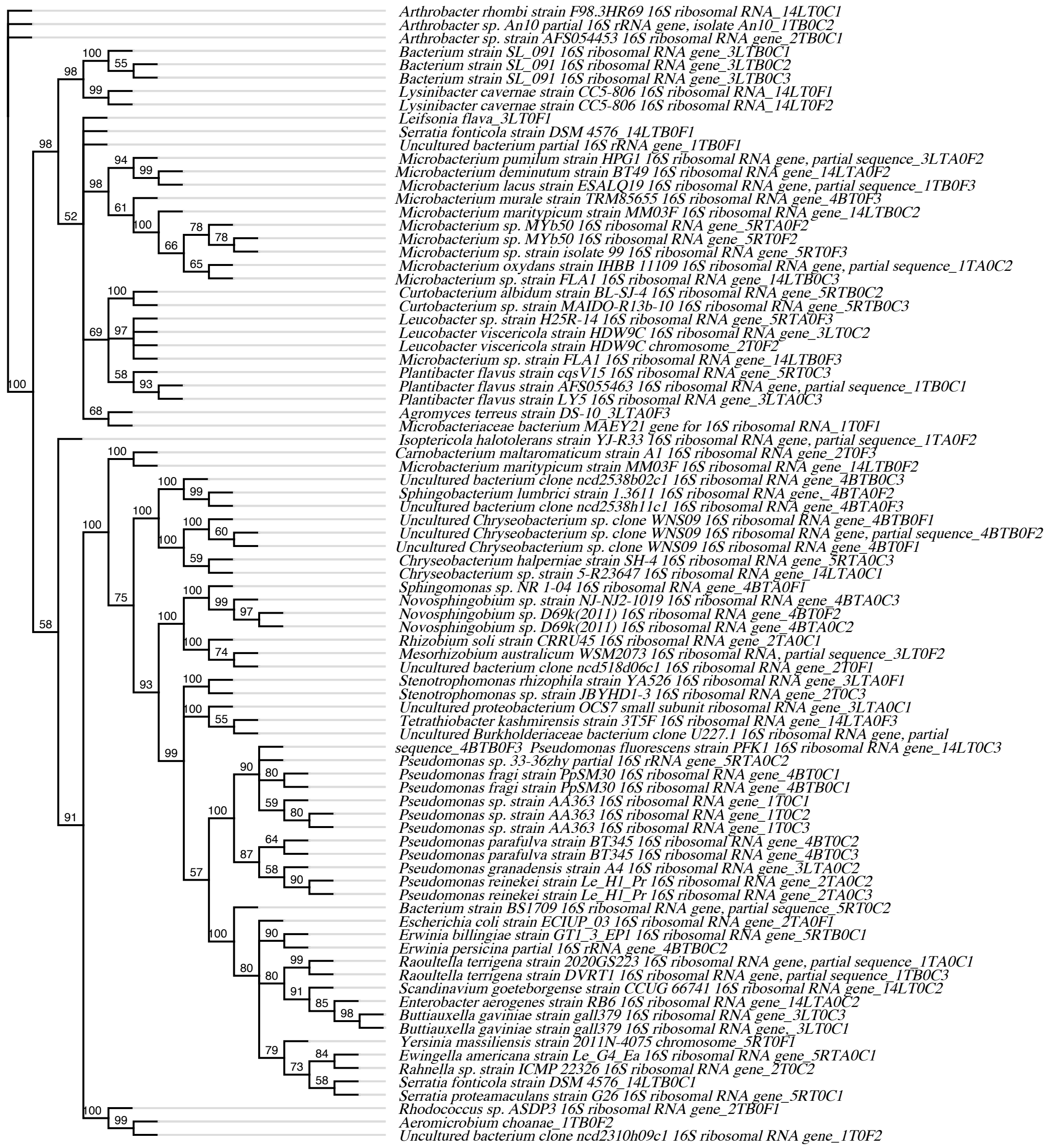

Fig. S1. The sequences were aligned with Geneious and identified using the RDP2 database online identification. The phylogenetic tree was reconstructed using the neighbor-joining method with the Tamura-Nei model of nucleotide substitution for focal isolates isolated from -7 weeks (pre-liming), 0 weeks (pre-liming) and 8 weeks (post-liming) time points. Numbers on branches represent bootstrapping support for that branch (per cent, 100 bootstraps).
