# Supplementary material for: Eco-evolutionary robustness of wild bacterial communities to experimental perturbation
Source: ISME J. 2025 Jul 22;19(1):wraf144. doi: 10.1093/ismejo/wraf144 (PMC12743297; doi:10.1093/ismejo/wraf144)
Supplement: SupplementaryFigure2_final_wraf144 [file supplementaryfigure2_final_wraf144.pdf]

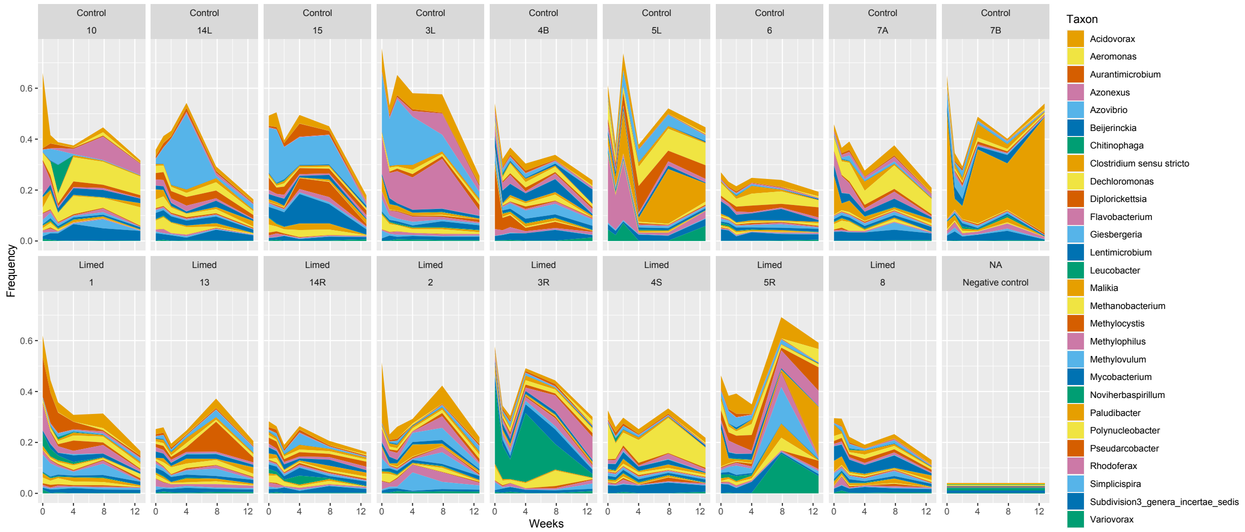

Fig. S2. The frequencies of 28 genera that had a frequency of at least 5% in any single sample. Top row = control tree holes, bottom row = limed tree holes. Colours recycle through a colour-blind palette, hence each used four times, but in the same order on the plots as in the legend.
