# Supplementary material for: Eco-evolutionary robustness of wild bacterial communities to experimental perturbation
Source: ISME J. 2025 Jul 22;19(1):wraf144. doi: 10.1093/ismejo/wraf144 (PMC12743297; doi:10.1093/ismejo/wraf144)
Supplement: SupplementaryFigure3_final_wraf144 [file supplementaryfigure3_final_wraf144.pdf]

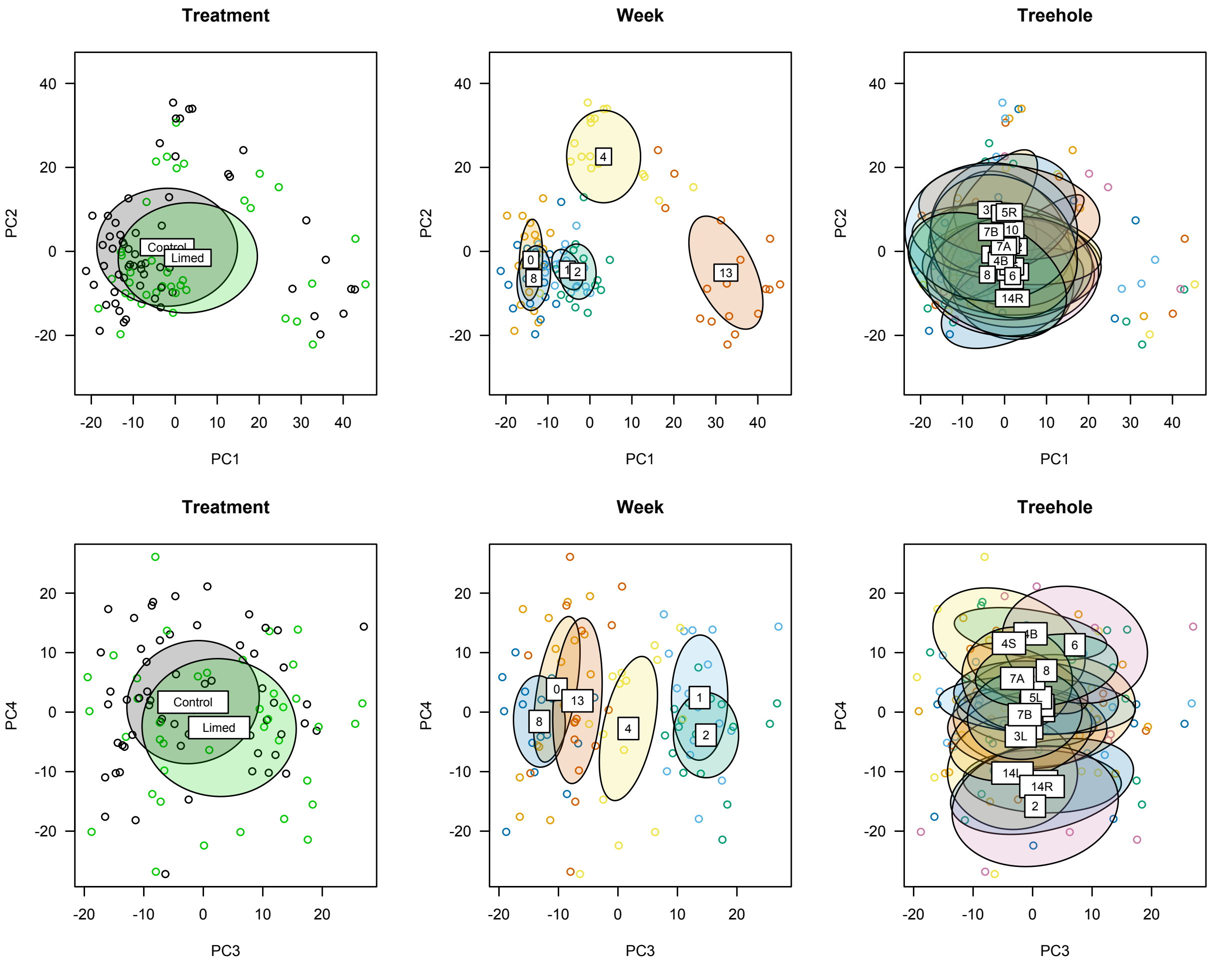

Fig. S3. Principal components analysis of centred log ratio transformed read counts, showing standard deviation ellipses around the centroid for each factor. Left column – Liming treatment, Central column – Week, Right column – Tree hole. Top row: PC2 versus PC1, Bottom row: PC4 versus PC3. PC1 to PC4 explained 18.5% of the variation in tree hole composition among samples.
