# Supplementary material for: Eco-evolutionary robustness of wild bacterial communities to experimental perturbation
Source: ISME J. 2025 Jul 22;19(1):wraf144. doi: 10.1093/ismejo/wraf144 (PMC12743297; doi:10.1093/ismejo/wraf144)
Supplement: SupplementaryFigure4_final_wraf144 [file supplementaryfigure4_final_wraf144.pdf]

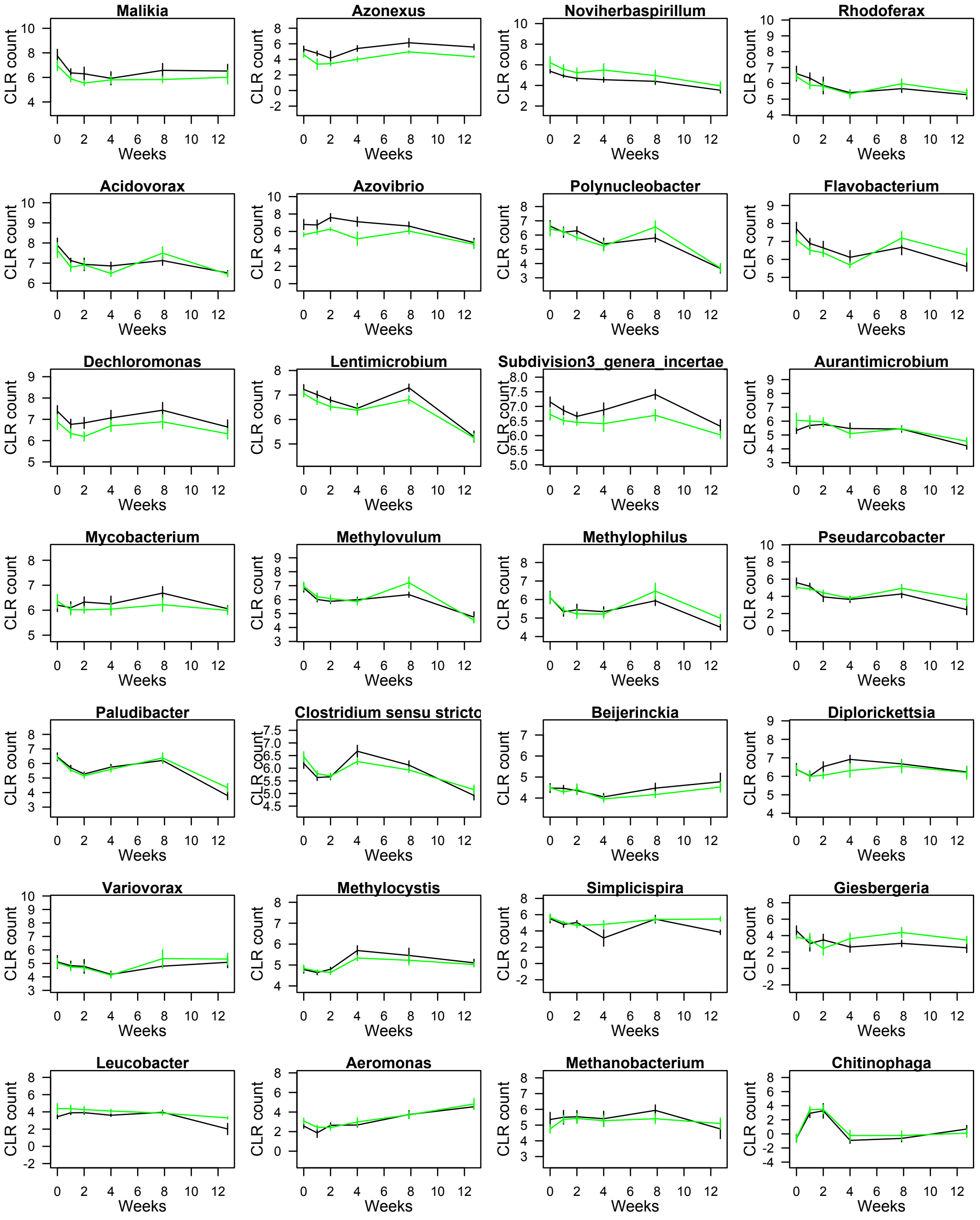

Fig. S4. The responses of the 28 genera with a maximum frequency >0.05 across samples to the liming treatment (black = control, green = limed) and over time (weeks). Mean and standard errors of the mean for each treatment and time combination are shown.
