# Supplementary material for: Eco-evolutionary robustness of wild bacterial communities to experimental perturbation
Source: ISME J. 2025 Jul 22;19(1):wraf144. doi: 10.1093/ismejo/wraf144 (PMC12743297; doi:10.1093/ismejo/wraf144)
Supplement: SupplementaryFigure5_final_wraf144 [file supplementaryfigure5_final_wraf144.pdf]

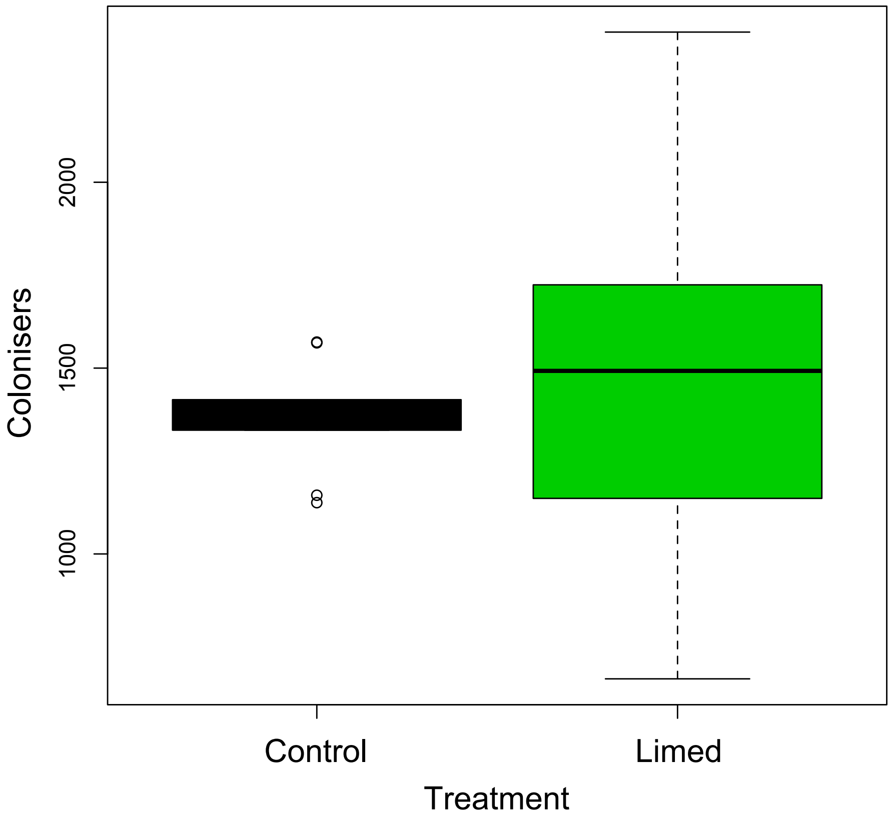

Fig. S5. Boxplot of the number of ASV colonisers (defined as ASVs that were absent at time zero and appeared at later timepoints and remained) in control and limed tree holes. The box shows the interquartile range, the bar shows the median, the whiskers show the most extreme data point no more than 1.5 times the interquartile range.
