# Supplementary material for: Eco-evolutionary robustness of wild bacterial communities to experimental perturbation
Source: ISME J. 2025 Jul 22;19(1):wraf144. doi: 10.1093/ismejo/wraf144 (PMC12743297; doi:10.1093/ismejo/wraf144)
Supplement: SupplementaryFigure6_final_wraf144 [file supplementaryfigure6_final_wraf144.pdf]

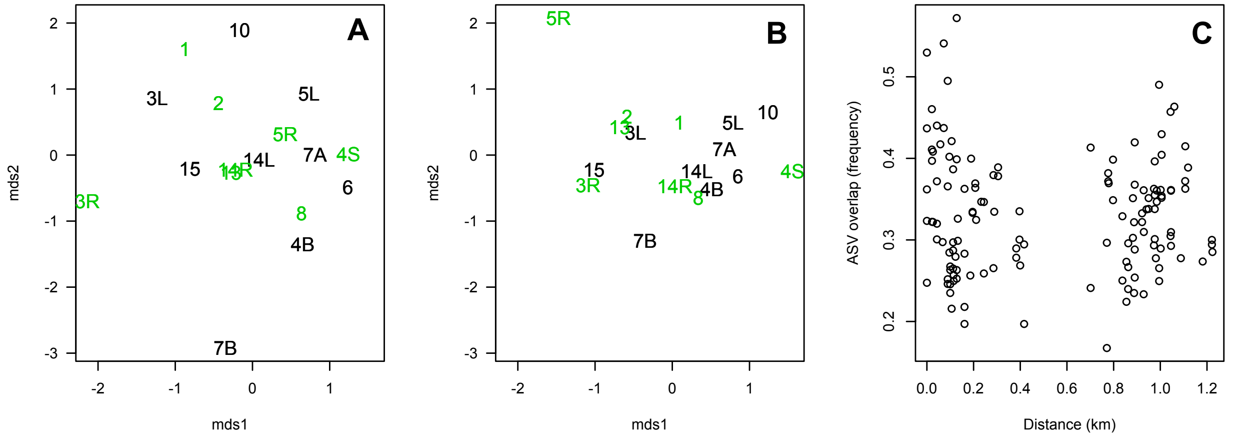

Fig. S6. A multidimensional scaling (MDS) plot of tree hole samples at **A**) week 0 and **B**) week 8 based on the degree of overlap of Amplicon Sequencing Variants (ASVs). Overlap was measured as the sum across ASVs of the minimum frequency between the two samples for each ASV. Then 1 – sharing was used as the input measure of "distance" for MDS. **C**) Pairwise overlap at week 0 plotted against geographic distances, each point represents a pair of tree holes.
