# Supplementary material for: Eco-evolutionary robustness of wild bacterial communities to experimental perturbation
Source: ISME J. 2025 Jul 22;19(1):wraf144. doi: 10.1093/ismejo/wraf144 (PMC12743297; doi:10.1093/ismejo/wraf144)
Supplement: SupplementaryFigure7_final_wraf144 [file supplementaryfigure7_final_wraf144.pdf]

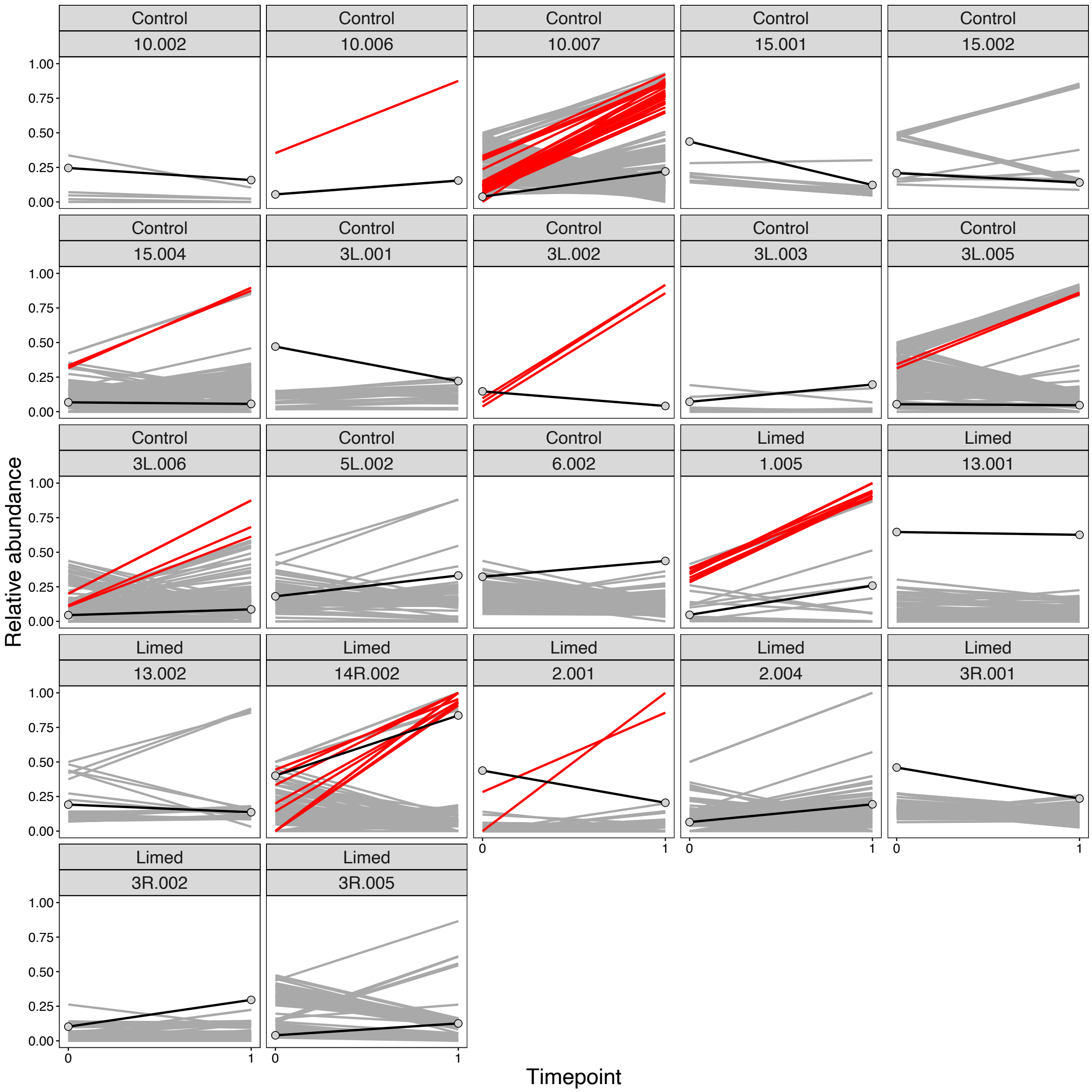

Fig. S7. Comparison of MAG abundance and sequence variants within each MAG. Each panel represents one of the 22 MAGs used for variant analysis, with the tree hole conditions and the identifier for the MAG in the panel title. Open points joined by a line show the relative abundance of the given MAG compared to the other MAG assemblies within the corresponding tree hole. Grey lines show changes in the frequency of all high-quality variants detected in that MAG. Red lines highlight "sweeping" variants – those with a change in relative abundance >0.5, starting with <25% abundance or finishing with >75% abundance.
