# Supplementary material for: Eco-evolutionary robustness of wild bacterial communities to experimental perturbation
Source: ISME J. 2025 Jul 22;19(1):wraf144. doi: 10.1093/ismejo/wraf144 (PMC12743297; doi:10.1093/ismejo/wraf144)
Supplement: SupplementaryFigure8_final_wraf144 [file supplementaryfigure8_final_wraf144.pdf]

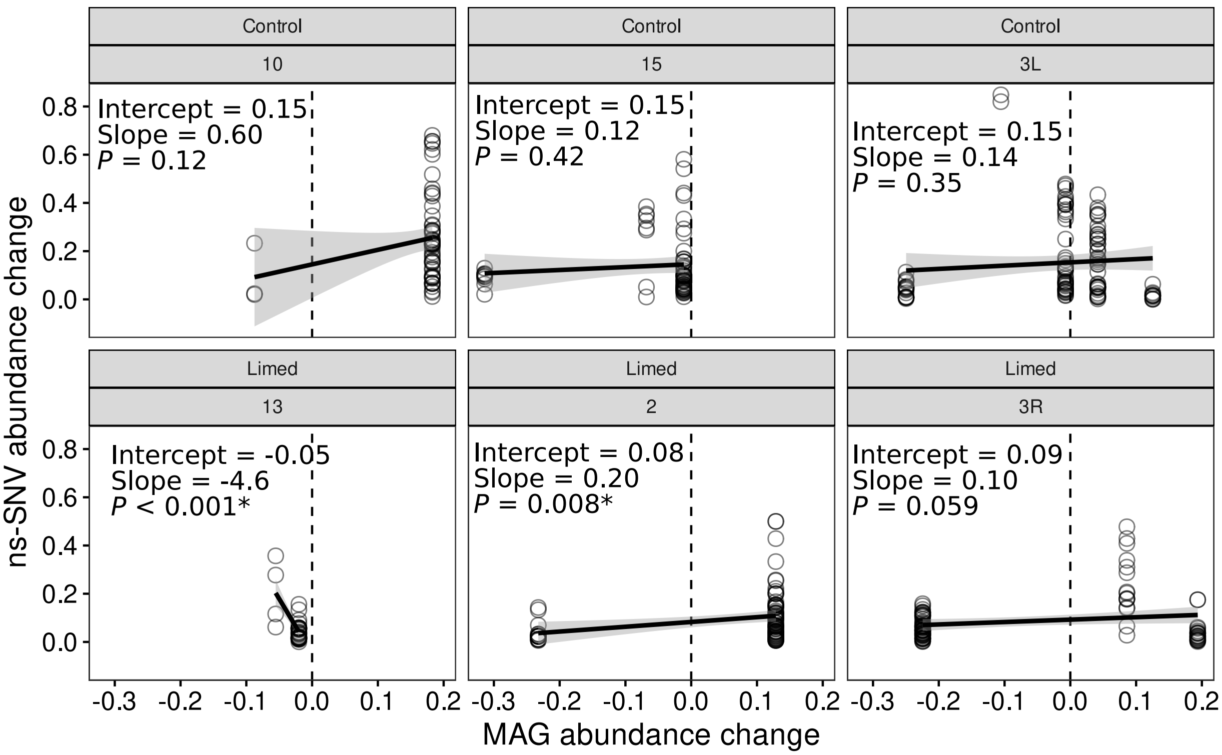

Fig. S8: Inter- vs intra-specific sorting by community. These are the changes in ns-SNV abundance regressed against the changes in abundance of MAGs they are found on, shown for each community (tree hole) with >1 MAG analysed. Dashed line marks zero MAG abundance change, delineating decreases (negative) and increases (positive) in MAG abundance between the timepoints. * = $P < 0.05$. Five out of six slopes are positive, but these are mainly non-significant ($P >= 0.05$).
