# Supplementary material for: Eco-evolutionary robustness of wild bacterial communities to experimental perturbation
Source: ISME J. 2025 Jul 22;19(1):wraf144. doi: 10.1093/ismejo/wraf144 (PMC12743297; doi:10.1093/ismejo/wraf144)
Supplement: Supplementary_information_wraf144 [file supplementary_information_wraf144.docx]

**Supplementary text S1**

*Justification of interaction coefficient*

We used time shift assays and growth on spent media to assess whole-community shifts in phenotypic characteristics. For example, a community *i* can be considered to use a set of resources when growing on fresh beech tea that leads to its growth to a value of *x_i_*. Similarly, community *j* can be considered to use a set of resources when growing on fresh beech tea that leads to its growth to a value of *x_j_*. Now consider that community *i* grows on media that has already been grown on by community *j*, attaining a growth of *x_ij_*. Prior growth of community *j* can either have a negative effect on growth of community *i*, e.g. if it used up shared resources or produces bioactive molecules with a negative effect on community *i,* or it can have a positive effect, e.g. if it produces waste products that community *i* can grow on, detoxifies the environment, or produces stimulatory bioactive molecules. If we assume the effect of community *j* depends on the density that it grew to in the medium, then a suitable metric of its effect is (*x_ij_* - *x_i_*)/*x_j_*. Positive values indicate stimulation of growth of community *i* relative to its growth on fresh medium and negative values indicate inhibition of its growth.

*Estimation of effective population sizes and generation times*

We used the variation in nucleotide allele frequencies in our MAGs to estimate in-situ population sizes and generation times in order to evaluate our observations. First, we estimated the per-site mutation rate, Watterson’s *θ,* for each MAG using:

$$\theta= \frac{K}{a_{n}\cdot L}$$

where *K* is the number of segregating sites (SNVs) at the first time-point, *L* is the total length of the MAG in nucleotides and *a_n_* is the (*n*-1)^th^ harmonic number, i.e.:

$$a_{n}= \sum_{i=1}^{n-1} \frac{1}{i}$$

where *n* is the average read depth of the MAG. The effective population size, *N_e_*, is the size of an idealised population that would show the same amount of genetic drift as the realised population. Effective population sizes for cultured bacteria have been reported in the order of 10^8^-10^9^ [36], however we lack estimates from field samples. For haploid organisms, we can estimate *N_e_* as:

$$Ne= \frac{\theta}{2\mu}$$

where *µ* is the per nucleotide mutation rate (mutations per site, per generation). To calculate *N_e_* for each MAG, we estimated *µ* as the median of bacterial mutation rates reported in Bobay and Ochman, 2018 [36] (2.0 x 10^-10^). We used the median here as the estimated mutation rates were right-skewed to high values. Following Jonas *et al.* [37], we then calculated the standardised variance in SNV frequency, *F*, for each SNV using:

$$F= \frac{\left( x-y \right)^{2}}{x\left( 1-x \right)}$$

where *x* and *y* are the SNV frequencies at timepoints 1 and 2, respectively. For each MAG, we then calculated the mean *F* (*F̅*) from degenerate sites (synonymous mutations, i.e. changes in frequencies driven primarily by drift rather than selection). Finally, using the above estimates we can estimate the number of generations, *t*, that have occurred between two timepoints if the observed changes are purely due to genetic drift [37]. We calculated *t* for each MAG using:

$$t= -2N_{e}\ln\left( 1-\overline{F} \right)$$

Given the 56 days between our timepoints, we calculated the generation time (or doubling time), *T_d_*, for each MAG as:

$$T_{d}= \frac{56}{t}$$

These quantities can be converted into growth rates, *r_max_*, using:

$$r_{max}= \frac{\ln\left( 2 \right)}{T_{d}}$$
